# Ebola virus disease outbreak in Guinea: what effects on prevention of mother-to-child transmission of HIV services?

**DOI:** 10.1186/s12978-018-0502-y

**Published:** 2018-04-10

**Authors:** Niouma Nestor Leno, Alexandre Delamou, Youssouf Koita, Thierno Souleymane Diallo, Abdoulaye Kaba, Therese Delvaux, Wim Van Damme, Marie Laga

**Affiliations:** 1 Bureau de Stratégie et de Développement du Ministère de la Santé, Conakry, Guinea; 2 Chair de Santé Publique de l’Université Gamal Abdel Nasser de Conakry, Conakry, Guinea; 3 Centre National de Formation et de Recherche en Santé Rurale de Maferinyah, Forécariah, Guinea; 4 Department of Public Health, Institute of Tropical Medicine, Antwerpen, Belgium; 5 Programme National de Prise en Charge Sanitaire et de Prévention des IST/VIH/Sida (PNPCSP) du Ministère de la Santé, Conakry, Guinea; 6 Secrétariat Exécutif du Comité National de Lutte contre le Sida (SECNLS), Conakry, Guinea

**Keywords:** Ebola, Effects, PMTCT, Health system, Guinea

## Abstract

**Background:**

An unprecedented epidemic of Ebola virus disease (EVD) affected Guinea in 2014 and 2015. It weakened the already fragile Guinean health system. This study aimed to assess the effects of the outbreak on Prevention of Mother-to-Child Transmission of HIV (PMTCT) services in 2014.

**Methods:**

We conducted a cross-sectional retrospective study. Data was collected from 60 public health centers (30 in the EVD affected areas and 30 in the unaffected areas). The comparison of PMTCT indicators between the period before Ebola (2013) and during Ebola (2014) was done using the t- test for the means and the Chi-square test for the proportions.

**Results:**

This study showed a substantial and significant reduction in the mean number of antenatal care visits (ANC) in the affected localities, 1617 ± 53 in 2013 versus 1065 ± 29 in 2014, *p* = 0.0004. This would represent 41% drop in health facilities’ performance. On the other hand, in the unaffected localities, the fall was not significant. The same observations were made about the number of HIV tests performed for pregnant women and the number of HIV positive pregnant women initiating ARVs. The study also noted an increase in the proportion of women tested HIV+ but who did not receive ARVs (12% in 2013 versus 44% in 2014) and HIV+ pregnant women who delivered at home (18% in 2014 versus 7% in 2013).

**Conclusion:**

This study showed that PMTCT services, which are one of the key services to improve maternal and child health, were affected in Guinea during this Ebola outbreak in 2014 compared to 2013.

## Plain English Summary

### Why was this study conducted?

This study aimed to assess the effects of the outbreak on PMTCT services in Guinée, in 2014.

### How was the study conducted?

We assessed changes in PMTCT indicators during the first year (2014) of the epidemic. We have included 60 public health centers, including 30 in affected areas and 30 in unaffected areas. The data collected covers the period from January 1, 2013 to December 31, 2014. The ANC and PMTCT registers were used for this collection. For each of the two areas, we compared the 2013 values to the 2014 values ​​to estimate the changes that occurred as effect of the outbreak.

### What was found in this study?

In 2014, the number of pregnant women attending ANC visits fell by 41% in the EVD affected localities, compared to only 7% in the unaffected localities.

All PMTCT indicators fell sharply during the 2014 epidemic compared with 2013. This decline was statistically more pronounced in Ebola-affected areas than in non-affected areas.

### What have we learned?

The weak capacity of the health system to respond to emergencies while continuing the provision of routine services and the mistrust between health care workers and communities contributed to low utilization of health services during this epidemic. It’s important to continue strengthening the Guinean health system to make it more resilient to mitigate the long-term effects of the epidemic and prepare it for future health crises. To this end, special emphasis should be placed on human resources for health.

## Article summary

### Strengths

- The comparison of indicators before and during the epidemic, as well as in Ebola-affected and unaffected areas, allow for a better estimate of the effect of the epidemic on PMTCT services;
- The study included PMTCT centers that did not have interruptions in supply (and therefore services) on a monthly basis of 5 or more days in 2013 to account for biases related to the unavailability of supplies.

### Limitation

- The use of routine data could include under-reporting bias because of the lack of regular monitoring of management tools.
- The population coverage could not be calculated because denominator data were no available. This is because the estimate of the “Spectrum” software used in Guinea could not provide disaggregated target values ​​by region, district, or heath facilities;
- Lack of information on how the pregnant women perceived the EDV epidemic situation and on the outcome of exposed children lost to follow-up.
- Retrospective data collection (design used in this study) could be one of the limitations of this study due to biases related to the management of data sources (e.g. registry) and the problem of reporting (under or overreporting).

## Background

### Introduction

An unprecedented epidemic of the Ebola virus disease (EVD) affected mainly three countries in West Africa, including Guinea, Sierra Leone and Liberia in 2014 and 2015. As of 17 January 2016, the cumulative number of cases in these three countries was 28,638, of which 11,316 died [1], representing a 39.51% lethality. This epidemic remains the largest in the world in terms of the number of cases, deaths and geographical distribution since the causative virus was isolated in 1970 [2].

In Guinea, the EVD outbreak experienced three major waves of transmission in 2014. The first two were located in Conakry and some prefectures of the forest region and the third was the phase of intense transmission at national level [3].

As of December 29, 2015, 3804 Ebola cases were reported in Guinea. Of these, there were 2536 deaths, a fatality rate of 66.6% [4]. In addition, it should be noted that more than 70% of country’s total Ebola cases occurred in 2014. Also, the highest number of human-to-human transmissions of the Ebola virus occurred in 2014 compared to 2015 [5]. 148 Ebola cases were confirmed among Health Care Workers as of 28 December 2014, with a fatality rate of 58.78% [5].

These alarming statistics suggest that the Ebola epidemic had probably significant indirect effects beyond the mortalities and morbidities it caused in the social and development sectors of affected communities between 2014 and 2015 [3]. The scale of the epidemic further affected the fragile health systems in the three most affected countries (Guinea, Liberia and Sierra Leone), in a way that vital and limited resources had to be reallocated to cope with the crisis, reducing the ability of countries to manage health problems [2, 6]. One aspect of the health system that could be hardly hit was the provision of basic health care services [3].

Plucinski et al., in their study conducted in 2014 noted a steady decrease of 11% in the use of health services for malaria at the operational level in Guinea during the Ebola period (2014) compared to the year before the outbreak (2013). They also noted a 15% decrease in the number of malaria cases reported in these structures [3].

It should also be noted that given the high maternal and infant mortality ratio that existed well before Ebola [7, 8], disruption of health services could be particularly damaging to maternal and child health services. A study conducted by Camara et al. [9] in 2016 showed a 51% decrease in the use of family planning services during the Ebola period in Macenta health district (Guinea) compared to the period before Ebola. The same study reported a significant 41% decrease in the number of women visiting health facilities for antenatal care [9]. Because PMTCT services are integrated into the minimum package of primary health care in Guinea, it would be expected that these services would be similarly affected by the outbreak.

Prevention of vertical HIV transmission is one of the strategies to eradicate HIV worldwide by 2030 [10, 11]; and one of the priorities of Guinea’s national AIDS strategy [12]. Many activities under this strategy occur in health care settings. Therefore, any reduction in the use of these services [13, 14], due to the EVD outbreak, could negatively impact the achievement of national targets for reducing vertical transmission of HIV and de facto reducing maternal mortality and child care in Guinea.

The purpose of this study was to describe and analyze the effects of the EVD outbreak in 2014 on PMTCT service provision in Guinea.

### Country general context

Guinea is located in West Africa with a population estimated at 11.7 million inhabitants in 2014, of whom 52% are women [15] and more than half (65%) of this population lives in rural areas. The country is one of the poorest countries in the world with 55.2% of the population living in poverty [7]. The health status of the population remains worrying, with high rates of maternal and infant mortality and high prevalence of certain diseases such as malaria, and adding to these are emerging and re-emerging diseases such as Ebola (2013–2015). Maternal mortality ratio increased from 980 to 724 per 100,000 live births between 2005 and 2012 [7].

In the interim report of 2016 multiple indicators clusters survey (MICS 2016) found a maternal mortality rate of 550 per 100,000 live births in Guinea, suggesting that Guinea remains among the countries with high maternal mortality rate in Africa [8]. In addition, the health status of children under 5 remains a concern. Children in this age group are among the most affected by malaria, which is the leading cause of morbidity and mortality in Guinea [7, 8]. In this age group (children under 5 years), the mortality rate before the first birthday is 88 per 1000 live births [8]. This situation could be explained by the weakness of Guinea’s traditional health care system, which is based on primary health care [16]. The system currently faces many challenges, including a low coverage of human resources for health (0.5 health personnel per 1000 inhabitants in 2016 for a norm of 2.3 per 1000 inhabitants), low immunization status in children (only 26% of children aged 12–23 months are fully immunized) and a concurrent high out of pocket payment for health expenditure (62% in 2014) [16].

### HIV and PMTCT care in Guinea

In Guinea, the HIV epidemic is of generalized type with an estimated national prevalence of 1.7% among people aged 15 to 49 years in 2012 [7]. According to recent estimates from UNAIDS, about 120,000 people were living with HIV in Guinea in 2014 4600 people died as a result of this disease [17].

The HIV prevalence is higher among women (2.1%) than men (1.2%) of the same age group (15–49 years) [7]. This disparity is more pronounced among pregnant women aged 15–49, whose HIV prevalence was 2.5% in 2008 and 3.56% in 2015 [18, 19]. According to estimates of the national AIDS control program, in 2014, 14% of new infections occurred among children, with 64% of them relating to vertical transmission (mother-child) [12]. The country’s PMTCT program was initiated in 2003 with seven pilot sites, including five in Conakry and two in Forécariah. A Scaling-up Plan was developed in 2008. A National Plan to Eliminate Mother-to-Child Transmission of HIV (e-PMTCT) was also developed in 2012 [20]. The current treatment regimen, in the context of PMTCT, is based on triple therapy known as “Option B+”, as recommended by the World Health Organization [21, 22]. It is initiated immediately after the diagnosis of HIV infection and continues for the rest of the mother’s life. Strategies identified by the national AIDS program to achieve the goals of eliminating mother-to-child transmission of HIV in Guinea by 2017 include lifelong administration of antiretroviral to HIV-positive pregnant women (Option B+), decentralization of PMTCT services to peripheral health facilities, use of community health workers to engage target populations at PMTCT sites, active search for those lost to follow-up, and use of blotting papers to collect and transport blood samples for the virological diagnosis of HIV [12]. These activities take place in health centers providing ANC services. The geographical coverage of public facilities that have integrated PMTCT activities remains low (65% of the existing 453 facilities in 2016) [23]. In 2013, there were already significant PMTCT gaps to be addressed in Guinea before the EVD outbreak occurs in 2014. The epidemic might have therefore aggravated these already existing gaps.

## Methods

### Study’s type

This was a retrospective cross-sectional evaluative study using routine data from public health facilities.

### Study population

The study targeted three categories of users of maternal and child health services including pregnant women, HIV-positive pregnant women and children born to HIV-positive mothers enrolled in public health centers providing prenatal and PMTCT services from 1 January 2013 to 31 December 2014.

We chosed 2014 as the year of comparison because the epidemic was not yet widespread in the country compared to 2015 and thus, could allow good comparison of different geographical areas.

The study included a representative sample of health facilities from across the country. Due to the wide disparity in the reporting of EVD cases, we divided the country into two strata. Stratum 1 consisted of the five most EVD affected health districts in 2014 and included Gueckédou, Macenta, Nzérékoré, Coyah and Conakry. Stratum 2 consisted of five randomly selected health districts among the districts not affected by the EVD in 2014. It included the health districts of Labe, Mamou, Boké, Kankan and Siguiri. This stratification was done to allow for comparisons, not only between the periods before (2013) and during (2014) the EVD outbreak in the same area, but also among the EVD affected and non-affected localities. Within each of the two strata, a random selection of 30 health centers offering both antenatal care and PMTCT services was carried out [24].

The selection of the study sites was conducted in steps. Step 1 included the establishment of the exhaustive list of health centre’s offering both ANC and PMTCT services by stratum. Step 2 included the identification of ANC/PMTCT sites that did not experience PMTCT services disruption for more than 5 days per month in 2013. Step 3 included the random selection of 30 ANC/PMTCT sites in each of the two strata, using the list established in step 2.

Computer random generated numbers were used for the different selections. When a selected site did not meet the selection criteria, it was replaced by the one immediately following in the sampling frame, and so on.

### Data collection

Antenatal care (ANC) and Prevention of Mother-to-Child Transmission of HIV (PMTCT) registers of the included public health facilities constituted the source for data collection.

A pre-elaborated survey questionnaire was used for data collection. The data collectors were selected from the national STI/HIV/AIDS prevention and management program (Programme National de Prise en Charge Sanitaire et Prévention des infections Sexuellement Transmissible et le Virus de l’Immunodéficience Humaine, (PNPCSP/IST/VIH) and the National AIDS Control Committee (Comité National de Lutte contre le Sida, CNLS) in Guinea.

They were selected based on their experience and knowledge of interventions to prevent mother-to-child transmission. Data collection was collected retrospectively. The approach was motivated by the low completeness of the statistical reports submitted to the national health information system and the results of previous evaluations showing discrepancies between data reported by health facilities and the actual data captured from facility registers [25]. The study focused on the PMTCT cascade. To this end, the following data was collected: 1) the number of pregnant women receiving ANC, representing pregnant women who made their first ANC visit for the current pregnancy and who were registered in the CPN registers; 2) the number of pregnant women tested for HIV which includes pregnant women who made their first ANC visit and received counselling about HIV testing, were screened for HIV and were registered in PMTCT registers; 3) the number of HIV+ pregnant women, representing women seen for their first ANC visit who were tested HIV positive and were registered in the PMTCT registers; 4) the number of HIV-positive pregnant women on ARVs, including HIV-positive pregnant women who initiated ARV treatment in PMTCT services and who were registered in PMTCT registers; 5) the number of exposed children on ARVs, representing infants born to HIV-positive mothers receiving antiretroviral prophylaxis for PMTCT; 6) the number of HIV+ pregnant women who gave birth at home, representing HIV-positive pregnant women registered in health facilities for PMTCT monitoring but who gave birth outside a health facility.

### Data analysis

Data was analyzed using the EPI Info 3.5.4 software (CDC Atlanta, USA). The data of this study focused on the overall performance of health centers offering prevention of mother-to-child transmission services. After entering all the data into an excel database, we performed a quality check as follows: (i) a complete check of all the values ​​of the study variables in the database to check for missing values and outliers and make necessary corrections and, (ii) quality check of a sample of the data collection cards to make comparisons between the database and paper copies.

The comparison covered two periods: period 1 from January 1 to December 31, 2013 (Pre-Ebola period) and period 2 from January 1 to December 31, 2014 (the first year of the Ebola epidemic in Guinea).

We used mean and standard deviations (SD) to examine the differences between absolute values. The t-test was used for normal distributions and the Wilcoxon test for asymmetric distributions. The absolute difference in performance between 2014 and 2013 was calculated for each stratum. We used the Chi-square test (χ2) to compare proportions. The difference was considered statistically significant for values ​​of *p* < 0.05.

Three main proportions were calculated including (i) the proportion of HIV-positive pregnant women on ARVs, obtained by dividing the number of HIV-positive pregnant women who initiated ARV treatment during our study period by the number of all pregnant women tested HIV-positive during our study period; (ii) the proportion of exposed children on ARVs, obtained by dividing the number of children born to HIV-positive mothers who received ARV prophylaxis by the number of pregnant women tested positive during our study period; and (iii) the proportion of HIV-infected pregnant women giving birth at home, obtained by dividing the number of HIV-positive pregnant women who gave birth at home by the number of pregnant women tested positive during the study period.

These proportions are based solely on the performance of the sites and not on the coverage of the population. This is because of the lack of available data that could provide disaggregated target values ​​by region, district, or care structure. The estimate of the “Spectrum” software used in Guinea only gives nationwide estimates and projections.

## Results

A total of 60 public health centers offering both ANC and PMTCT services were included in the analysis.

Among facilities located in EVD affected areas, there was a significant reduction in the mean number of pregnant women attending ANC visits, 1617 ± 53 in 2013 compared to 1065 ± 29 in 2014 (*p* = 0.0004. In non EVD affected localities, however, the decrease was not statistically significant (from 1817 ± 331 in 2013 to 1689 ± 280 in 2014 with, *p* = 0.5696 (Table 1). The same findings were made for the number of pregnant women tested for HIV, the number of HIV-positive pregnant women on ARVs and the number of exposed children who received prophylactic ARVs (Table 1).

**Table 1:** PMTCT indicators comparison by year and by Ebola areas, 2014, Guinea

INDICATORS	Ebola areas affected means±SD			Ebola unaffected areas means±SD		
	2013	2014	*P*-value	2013	2014	P-value
Pregnant women receiving ANC	1617 ± 53	1065 ± 29	0.0004	1817 ± 331	1689 ± 280	0.5696
Pregnant women tested for HIV	1460 ± 266	717 ± 140	0.0000	1622 ± 247	1379 ± 212	0.1556
Pregnant women HIV+	44 ± 13	31 ± 9	0.1292	27 ± 7	27 ± 6	0.9723
HIV-positive pregnant women on ARVs	28 ± 6	13 ± 3	0.0000*	24 ± 7	22 ± 5	0.6513
Exposed children receiving prophylactic ARVs	22 ± 5	6 ± 1	0.0000*	17 ± 5	16 ± 3	0.6020

*=no - parametric test

Number of sites: 30 in the Ebola zone and 30 in the non-Ebola zone

**Fig. 1:**
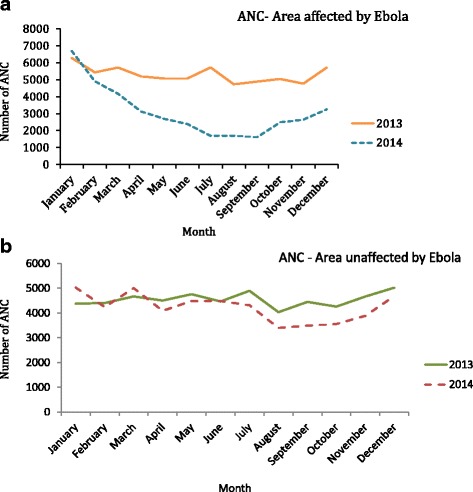
**a** Comparison of the monthly change in the number of antenatal visits between 2013 and 2014 in 30 PMTCT facilities in EVD affected areas in 2014, Guinea. **b** Comparison of the monthly change in the number of antenatal visits between 2013 and 2014 in 30 PMTCT facilities in non EVD affected areas in 2014, Guinea

Figure 1a presents the monthly trend of PMTCT activities in EVD affected localities in 2014. It appears that the monthly number of pregnant women attending ANC visits was more or less stable in 2013 with a peak in July. However, in 2014, the trend was modified. The fall began between February and March, peaking between July and September 2014 with a slight increase between October and December 2014 (Fig. 1a). In contrast, in unaffected localities, the monthly trends of the mean number of ANCs visits in 2013 and 2014 are almost similar (Fig. 1b).

Differences in the performance of health facilities between 2013 and 2014 in terms of PMTCT services are presented in Table 2. In 2014, the number of pregnant women attending ANC visits fell by 41% in the EVD affected localities, compared to only 7% in the unaffected localities. The fall in the number of HIV+ pregnant women who received ARVs was 55% in the EVD affected area compared to only 8% in the unaffected area. Similar trends were observed for two other indicators including the number of pregnant women tested for HIV (with a 53% drop) and the number of exposed children receiving prophylactic ARVs (with a 69% drop) (Table 2). (In EVD affected areas, the changes were greater during the third quarter of 2014 compared to the same period in 2013. Regarding the number of HIV-infected pregnant women on ARVs, the fall did not change between the different quarters of 2014, except for the first quarter (Table 2).

**Table 2:** Changing in indicators by quarter and by the EVD status of the study areas, Guinea

	Area affected by Ebola					Area not - affected by Ebola				
	Q1	Q2	Q3	Q4	Global	Q1	Q2	Q3	Q4	Global
Pregnant women receiving ANC	−9%	−47%	− 67%	−46%	−41%	6%	−5%	−16%	−13%	−7%
Pregnant women tested for HIV	−8%	−69%	− 77%	−62%	− 53%	11%	− 14%	−31%	−25%	− 15%
Pregnant women HIV+	−15%	−19%	−29%	−52%	−28%	23%	16%	−18%	− 19%	−1%
HIV-positive pregnant women on ARVs	−14%	− 71%	−68%	−77%	−55%	22%	0%	−27%	−23%	−8%
Exposed children receiving prophylactic ARVs	−28%	−86%	− 86%	−89%	−69%	25%	−16%	−26%	−15%	−9%

*Q* Quarter, *Q1* January to March, *Q2* April to June, *Q3* July to September, *Q4* October to December

**Fig. 2:**
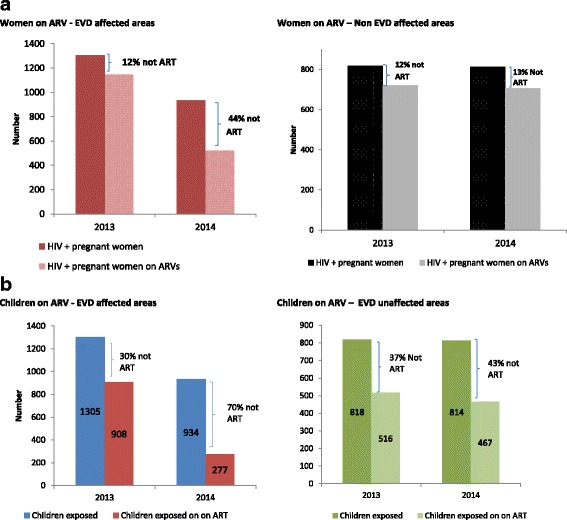
**a** Comparison of the proportion of HIV-positive pregnant women who initiated ARVs between 2013 and 2014, stratified by EVD status of the study areas, Guinea. **b** Comparison of the proportion of HIV-positive pregnant women who were put on ARVs between 2013 and 2014, stratified by the EVD status of the study areas, Guinea

**Fig. 3:**
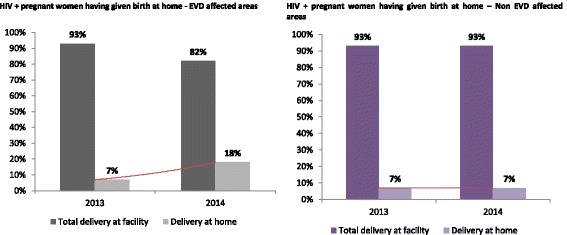
Comparison of the proportion of HIV-infected pregnant women who gave birth at home (outside a health facility) between 2013 and 2014, stratified by the EVD status of the study areas, Guinea

Figure 2a compares the proportion of pregnant women tested positive for HIV who did not receive ARVs in order to reduce the risk of mother-to-child transmission of HIV, but also to improve their own health status. This proportion increased during the first year of the Ebola outbreak in the EVD affected areas, from 12% in 2013 to 44% in 2014, *p* < 0.05. However, in the unaffected areas, there were no statistically significant changes (12% in 2013 compared to 13% in 2014, *p* = 0.427) (Fig. 2b). Figure 3 shows that the proportion of HIV+ pregnant women who gave birth at home increased significantly during the EVD outbreak (7% in 2013 versus 18% in 2014) in the affected areas, *P* < 0.0001.

## Discussion

Our study shows that in Guinea, the use of PMTCT services significantly decreased during the EVD outbreak (2014) compared to the period before the EVD (2013). In addition, our results show that the decrease in the use of PMTCT services was significantly more pronounced in the EVD affected areas compared to the areas not affected by the EVD in 2014.

Our results corroborate those reported by Delamou et al., in a study on the effects of the EVD on maternal and child health services in the forest region of Guinea [26]. The authors noted a significant reduction in the number of ANC visits and institutional deliveries during the epidemic compared to the pre-epidemic period. Similarly, they reported a significant drop in the number of immunized children and expressed concerns about the post-epidemic trends that were still below figures of the pre-epidemic period. Despite the methodological differences between our two studies (completeness of data in their study and purposive sampling in ours), we reached the same conclusions. At the same time, this could also mean that PMTCT services would not probably regain the levels they had before the epidemic. Our results are also similar to the findings of a review of 22 studies conducted in 2016 by Ribacke et al., which reported a decrease in the number of ANC visits by 27% during the last six months of 2014 at national level in Sierra Leone and by 50% in Moyamba health district [13].

The significant drop in the number of ANC visits, pregnant women tested for HIV, and the number of HIV-positive pregnant women receiving antiretroviral therapy may be due to a several factors. The first is the fear of communities to contract EVD in health facilities where many health workers were already infected [24]. This fear resulted from mistrust between health service providers and the community ( [26]. This was the consequence of rumors around Ebola. One of the rumors circulating said that there was acomplicity between the political authorities and the International Non-Governmental Organizations (NGOs) to invent this epidemic in order to attract external funding and make financial profits. The second factor, a direct consequence of contaminations (lack of knowledge of the transmission modes and low protection of health workers) and deaths recorded among health personnel, would be the desertion of health facilities by the staff [27]. As a result, the supply and quality of health services might have been reduced. The third factor is the temporary closure of some health facilities during the peak of the epidemic [3]. The fourth factor is about the reorientation of a large number of health workers and available resources to EVD response activities (epidemiological surveillance, contact tracing, awareness raising) deemed less risky and more financially profitable.

The reduction in the number of ANC visits might have resulted in reduced demand for HIV testing among pregnant women in health centers. In fact, some women, even those who attended ANC, have been able to refuse HIV testing because of rumors about EVD transmission by blood and needles. Even though this information has not been collected, it is possible that this situation has led to an increase in the rate of vertical transmission of HIV due to ignorance of the serological status of pregnant women and increased viral load in women living with HIV, known but poorly managed.

Leuenberger et al. [26] also observed a 46% decrease in the number of patients screened for HIV in 2014 in the Medical Center of the African Phil Mission in Macenta (one of the most EVD affected district in forest Guinea in 2014). In addition, a report by UNICEF on the impact of EVD in Sierra Leone mentioned that the use of PMTCT services in the country fell by 23% in 2014 compared to 2013 [28].

EVD has also disrupted the HIV supply and distribution system at the national level by reducing the access of pregnant women to ARVs. EVD occurred in a context where HIV product management was still centralized and where international technical assistance may have been lacking due to travel restrictions and border closures [13].

Our study also showed a significant increase in the proportion of HIV-positive pregnant women who gave birth at home and the high rate of loss to follow-up among exposed children. This situation could be explained by two factors. First, even after having followed the ANC visit, women would fear to be contaminated by the EVD during deliveries in health facilities that were stigmatized for Ebola. Second, the reorientation of many community mediators, usually in charge of follow-up, to the EVD response activities might have negatively affected PMTCT services outcomes. A high number of HIV patients lost to follow-up (18.4% at six months) was reported in the study by Leuenberger in Macenta district [29]. Similar figures were reported in the systematic review by Ribacke et al., which showed a significant increase (*p* < 0.001) in the number of HIV patients lost to follow-up at 3 months, between June and October 2014 in Liberia [13].This could have led to an increased risk of mother-to-child transmission of HIV (MTCT) due to increased numbers of unsafe home deliveries, jeopardizing the investments of countries and their partners in the reduction of maternal and infant mortality that were in place before the EVD.

Our study had some limitations, in particular the lack of information on the perception of pregnant women and the future of exposed children lost to follow-up. In addition, only sites offering both ANC and PMTCT services were included in the study. This does not provide information about those offering only ANC. In addition, the collection of routine data could include under-reporting bias because of the lack of regular monitoring of management tools as health care workers were occupied with the EVD response. Furthermore, the population coverage was not calculated because of the lack of target populations for study areas. Nevertheless, our study is one of the few studies that compared indicators before and during the EVD and compared indicators between EVD affected and non-affected areas in 2014. In addition, the study included PMTCT sites that did not experience stock rupture of more than five days a month in 2013 to account for biases related to unavailability of supplies.

## Conclusion

The present study estimated the collateral effects of the EVD outbreak on PMTCT services in Guinea. All PMTCT indicators experienced a drastic fall during the 2014 epidemic compared to 2013. This fall was more pronounced in EVD-affected areas compared to non-affected ones. Lost to follow-up and home deliveries (unsecured) increased significantly. The weak capacity of the health system to respond to emergencies while continuing the provision of routine services (resilience) and the mistrust between health care workers and communities contributed to low utilization of health services during this epidemic. It is important to continue strengthening the Guinean health system to make it more resilient to mitigate the long-term effects of the epidemic and prepare it for future health crises. To this end, special emphasis should be placed on human resources health.

## Abbreviations

ANC: Antenatal Care
ARVs: Antiretroviral
e-PMTCT: Eliminate Mother-to-Child Transmission of HIV
EVD: Ebola Virus Disease
HIV: Human Immunodeficiency Virus
IST: Infection Sexuellement Transmissible
Option B+: Antiretroviral therapy for all pregnant women tested positive for any immune status for life
PMTCT: Prevention of Mother-to-Child Transmission of HIV
PNCSP: Programme National de Prise en Charge Sanitaire et de Prévention des IST/VIH/Sida
SECNLS: Secrétariat Exécutif du Comité National de Lutte contre le Sida
Sida: Syndrome de l’Immunodéficience Acquise
UNICEF: United Nations Children’s Fund
VIH: Virus de l’Immunodéficience Humaine
